# Healthcare First: Delivering Immediate, Non-Contingent, and Coordinated Services Through the Street Medicine Model

**DOI:** 10.21203/rs.3.rs-8233628/v1

**Published:** 2026-02-12

**Authors:** Presleigh Beshirs, Siddhi Ganesh, Ricky Bluthenthal, Bradley Conner, Ashleigh Herrera

**Affiliations:** California State University Bakersfield; University of Southern California; University of Southern California; Colorado State University; California State University Bakersfield

**Keywords:** Street medicine, people experiencing homelessness, unhoused patients, healthcare delivery, harm reduction

## Abstract

**Background:**

People experiencing homelessness (PEH) face disproportionate morbidity and mortality due to structural barriers that restrict healthcare access. Conventional health systems often exacerbate these inequities through conditional treatment models requiring readiness, compliance, or abstinence. Street medicine - interdisciplinary teams delivering care directly in community settings - has emerged as a critical response. Despite its growth, limited qualitative research explores patient perspectives on how street medicine structures care to counter systemic exclusion. This study examines the experiences of unhoused patients receiving street medicine care in Bakersfield, California.

**Methods:**

Between March and May 2025, we conducted 10 semi-structured interviews with individuals receiving street medicine services in Bakersfield, California. Eligible participants were aged ≥ 18, English-speaking, and had received care within six months. Using a constructivist grounded theory approach, interviews were audio-recorded, transcribed, and analyzed iteratively in ATLAS.ti. Coding and memo writing were used to develop a conceptual model describing how street medicine structures healthcare delivery and engagement.

**Results:**

Participants described street medicine as characterized by three interrelated mechanisms: (1) immediate, non-contingent access fostering trust and engagement; (2) integration of harm-reduction principles across medical and behavioral services; and (3) embedded navigation reframing adherence as a system-supported process. Collectively, these processes aligned with a Healthcare First model that parallels Housing First by emphasizing immediacy, choice, and voluntary engagement.

**Conclusions:**

Street medicine reframed healthcare as a right rather than a conditional service, countering structural exclusion through accessibility, consistency, and dignity. Findings underscore the importance of institutionalizing Healthcare First principles to promote equity and continuity of care for PEH.

## 1. Background

### 1.1 Housing Crisis

The United States is facing an unprecedented affordable housing crisis, characterized by widespread housing insecurity, inadequate living conditions, and record levels of homelessness (Keene & Blankenship, 2023). Nearly one-third of Americans now spend 30% or more of their income on housing, putting stable and affordable housing increasingly out of reach (Mehdipanah, 2023). Between 2018 and 2024, homelessness rose by nearly 40% in the United States, including a 41% increase in unsheltered homelessness, with more than 771,000 individuals experiencing homelessness on a single night in 2024, of which 274,000 were unsheltered, marking the highest levels ever recorded (Colburn et al., 2024; Pillai et al., 2024).

### 1.2 Housing First (HF) Model

The Housing First (HF) model, developed by Dr. Sam Tsemberis and the Pathways to Housing program in New York City in 1992, is founded on the principle that housing is a basic human right (Tsemberis, 1999). The approach offers individuals experiencing chronic homelessness - particularly those with co-occurring mental illness or substance use disorders - immediate access to permanent, subsidized housing paired with supportive services, without requiring sobriety or treatment compliance (Tsai, 2020; Watson et al., 2017). Emphasizing consumer choice, autonomy, and harm reduction, HF recognizes that stability in housing provides the foundation from which recovery and behavioral change can occur (O’Campo et al., 2022).

Research demonstrates that HF is more effective than traditional “Housing Ready” models in reducing homelessness and improving housing stability (Goering et al., 2014; Tsemberis et al., 2004). Participants also experience reductions in emergency department visits, hospitalizations, and other crisis service utilization (Basu et al., 2012; Davidson et al., 2014). However, outcomes related to broader health improvement are mixed, and access to HF programs remains limited - the number of available units and services falls far short of the need, leaving many people unhoused and at continued risk for poor health and premature death (National Academies of Sciences, Engineering, and Medicine, 2018; Culhane et al., n.d.). This gap underscores the necessity of complementary, field-based approaches such as street medicine, which provide critical care and harm reduction services for those who remain outside the reach of housing-based interventions.

### 1.3 Healthcare Crisis

PEH face profound health inequities and disproportionately high disease burdens compared to the general population, driven largely by structural and social determinants of health such as unstable housing, poverty, and limited access to healthcare services (Lee et al., 2023). Substance use disorders are two to three times more prevalent among PEH (Pillai et al., 2025), with high rates of polysubstance use - particularly opioid-involved - contributing to the rising epidemic of overdose deaths (Fine et al., 2022). Approximately one in four PEH lives with a serious mental illness such as schizophrenia, bipolar disorder, or major depression (Pillai et al., 2025), while chronic diseases including hypertension, diabetes, COPD, cardiovascular disease, and untreated pain are also significantly overrepresented and often exacerbated by fragmented care (Bensken et al., 2021; HUD, 2012). PEH experience increased vulnerability to infectious diseases such as HIV, hepatitis C, and tuberculosis (Lui et al., 2020), with higher HIV prevalence rates than among housed populations (Arum et al., 2021; Stone et al., 2022). These overlapping conditions, compounded by delayed and inconsistent access to treatment, result in premature mortality rates three to ten times higher and a life expectancy nearly 30 years shorter than the general population, underscoring the urgent need for integrated, accessible, and equity-focused health interventions (Bedmar et al., 2022; Bishop et al., 2025).

### 1.4 Limitations in Treatment as Usual Approach to Healthcare among PEH

PEH face numerous structural and social barriers to accessing healthcare in traditional settings, including insurance status, lack of personal identification, lack of transportation, and apprehension of healthcare systems due to experiences with stigma and discrimination (eClinicalMedicine, 2023). Previous studies have documented that PEH reported experiences of discrimination as well as poor and biased treatment in primary, emergency, and acute care settings (Gilmer & Buccieri, 2020; Lauricella et al., 2025; Reilly et al., 2022; Wen et al., 2007). Given these barriers to care and their elevated disease burden, PEHs frequently utilize hospital emergency departments as their primary means to access healthcare services (Vohra, 2022). However, previous research has demonstrated that acute, emergency care settings are costly and ineffective in addressing PEH’s complex healthcare needs, resulting in a revolving door from the streets to the emergency department (Franco et al., 2021; HUD, 2012; Vohra et al., 2022).

### 1.5 Street Medicine

Street medicine is the practice of delivering medical care directly to people experiencing homelessness (PEH) where they live - on the streets, in encampments, under bridges, or in other unsheltered settings (National Health Care for the Homeless Council, n.d.). Established in the early 1990s (Withers, 2011; Feldman et al., 2023), the model has expanded to more than 150 programs across the United States (Medellin et al., 2024) and over 60 throughout California (Keck School of Medicine of USC, 2024). Teams typically include physicians, nurses, medical assistants, case managers, and behavioral health professionals (Perna et al., 2024). By bringing care directly to individuals excluded from traditional healthcare systems, street medicine addresses the structural, geographic, and interpersonal barriers that make medical care largely inaccessible for PEH (Medellin et al., 2023).

Street medicine programs are grounded in harm reduction principles that have been increasingly recognized as essential to medical care delivery more broadly (Hawk et al., 2017). These principles - humanism, pragmatism, individualism, and autonomy - are clearly espoused in the street medicine model. Guided by this philosophy, teams adopt a nonjudgmental, person-centered approach that emphasizes trust, patient choice, and shared decision-making. Access to medical care or material support is not contingent on abstinence, treatment participation, or compliance. In practice, these values translate into harm reduction–oriented practices such as the distribution of naloxone and safer use supplies, infection and wound care, and the creation of safe, stigma-free spaces where patients can disclose substance use without fear of judgment or denial of services. Through this approach, street medicine fosters relational continuity, empowerment, and engagement among patients who have long been excluded from traditional care.

This qualitative study explores patient perspectives on how these principles shape the effectiveness of street medicine in domains such as access to care, treatment adherence, and patient activation, and contrasts these experiences with traditional healthcare delivery in Bakersfield, California.

## 2. Methods

This research was conducted with PEHs (n = 10) who were receiving care from local street medicine teams located in Bakersfield, CA, from March 2025 to May 2025. All study participants provided verbal informed consent for study participation. The participants of this study were recruited through a convenience sampling strategy among people who were receiving services from two street medicine teams located in Bakersfield, CA, between March 2025 and May 2025. Participants were eligible to participate in this study if they were over the age of 18, had received street medicine services within the past 6 months, were residents of Bakerfield, and were English speakers. This population was deliberately selected for this study due to their poor healthcare access, due to geographic location, limited transportation means, and lack of proper identification needed to access healthcare services. To protect participant confidentiality and anonymity, we assigned pseudonyms to the participants.

We conducted 30- to 60-minute interviews with participants [Appendix A - Interview Guide]. Using a constructivist grounded theory approach, the research team followed an iterative process for data analysis (Charmaz & Thornberg, 2020; Charmaz, 2006), which is outlined in further depth in our previous publication (Beshirs et al., 2025). The research team reviewed the transcripts from the participant interviews, recorded initial analytic findings, and created a codebook with thematic codes [Appendix B - Codebook]. Codes were conceptually grounded through iterative analysis, memo writing, and comparison, and results were developed through the coding of blocks of texts across all interview transcripts using ATLAS.ti (Version 26) (ATLAS.ti Scientific Software Development GmbH, 2025). Through these analyses, we developed a conceptual model to describe participants’ experiences. We focused on findings pertaining to access to care, treatment adherence, and patient engagement and activation, which yielded four results.

## 3. Results

The analytic sample consisted of 10 street medicine patients residing in Bakersfield, California. The majority identified as female (n = 6; 60%) and Latine (n = 7; 70%). Most patients reported experiencing chronic unsheltered homelessness for over 12 months (n = 7; 70%), while three recently moved into transitional housing sites following extended periods of unsheltered homelessness. Despite being rehoused, these individuals continued to receive services from their street medicine team. All participants were actively engaged with one of two street medicine teams operating in Bakersfield, California.

The qualitative interview questions were designed to explore the acceptability of street medicine services, prompting patients to highlight the specific elements of this practice model that made it acceptable to them. We found that street medicine: 1) provided immediate and noncontingent access to healthcare, 2) integrated a harm reduction approach into the provision of healthcare, and 3) provided integrated case management services to support recovery and health goals, whereas the treatment as usual model stood in opposition to this practice framework.

### 3.1 Unhoused patients encounter institutional barriers to accessing traditional healthcare due to emphasis on adherence and consistency as prerequisites for continued care.

Patients described the ways in which traditional healthcare systems often systematically excluded unhoused patients by prioritizing adherence and consistency as prerequisites for continued care, relying on authoritarian decision-making, and placing the burden on patients to navigate fragmented and complex systems - without addressing their material needs or the structural barriers that prevent access to care. As a consequence, unhoused patients internalized their inability to enact health practices and routines to meet their health goals as personal failings rather than institutional decisions to explicitly and intentionally exclude unhoused people. In turn, this disempowerment led to an erosion of self-efficacy - including a sense of loss of agency, control, ability to make healthcare decisions, and enact health behaviors all of which were shaped by broader social and structural forces such as houselessness, poverty, and lack of healthcare access.

Patients, like Luis, described the significant executive and cognitive demands of navigating traditional health care systems. For Luis, the psychological stress of scheduling, attending, and understanding appointments ultimately led to disengagement from care.

“I sometimes get anxiety attacks; I haven’t had them in a while. When I did, it made it harder to go and see the doctor. It’s hard to make myself go and see the doctor and actually make the appointment… I never felt like they [traditional clinics] helped me with all the stuff [street medicine team] helps me with. Clinics and doctors can be very demanding; it would be stressful to make the appointments or try to understand what I needed to do when I’m not good at stuff like that.”

- Luis

Luis discussed the negative feedback loop in traditional health care settings, which reinforced his disengagement from healthcare systems. When he attempted to access care through traditional settings, he felt overwhelmed by the requirements to navigate his care independently. Despite the institutional failure to emphasize health literacy, provide care coordination, and account for the complex lived realities of unhoused patients (e.g. lack of consistent access to a cellphone or the internet, lack of transportation, lack of timekeeping devices), Luis attributed these institutional failures to his self, thereby internalizing them. Over time, this eroded his belief and ability to engage with the healthcare system. His compromised self-efficacy caused high levels of anxiety and psychological distress, further deterring him from reattempting to engage with his healthcare needs through traditional systems of care. Luis’ account highlights how the demands placed on unhoused patients by traditional health care systems exceed their capacity due to structural vulnerabilities and disempowerment.

Similarly, Aliyah discussed how the barriers she faced in traditional health care systems became internalized as feelings of being incapable of caring for her health, and implied that they were due to provider-driven negative perceptions of her. While unhoused, she often blamed herself for being unable to manage the demands of clinic-based care.

“I had pneumonia just a while back, and I needed to get my lungs checked. It was something I was supposed to be doing at the clinic, but I just didn’t keep up with it. Without [the street medicine team], I probably wouldn’t have gotten it checked again. They made sure I got my antibiotics and everything was good.”

- Aliyah

However, patients like Aliyah internalize these structural failures, such as lack of transportation and the need to participate in survival economies, contributing to her negative self-appraisal and compromised self-efficacy, leading to disempowerment and long-term disengagement from healthcare systems. This structural disempowerment delayed the treatment of her pneumonia, which could have led to her untimely death; however, she became connected to a street medicine team and finally received care designed for her.

Similarly, Robert explained that he disengaged from traditional health care and follow-up care because of both limited access to information and the absence of support to build health literacy. With the emphasis on adherence and service delivery model centered around housed populations with some level of material security, providers in traditional healthcare settings do not provide patient-driven care and fail to promote reciprocal learning and tailored messaging and interventions for unhoused patients.

“They [traditional clinics] would tell me I needed lab work and just expect me to get it done. But I didn’t know why I needed it or how to do it.”

- Robert

Robert went on to note that clinics often failed to account for the structural and contextual barriers he faced, including transportation challenges, concerns about the safety of his encampment sites and pets, and opportunity costs of attending appointments, highlighting the importance of integrated case management to facilitate healthcare engagement.

Similarly, other unhoused patients, like Alejandro, were unable to access traditional health care because these systems often require insurance and government identification.

“I had no documents [ID, health insurance] before I started seeing them [street medicine team], so when I wanted to work on some substance stuff, I wasn’t able to do that anywhere.”

- Alejandro

Alejandro’s underscored how traditional healthcare systems failed to account for the complex lived realities of unhoused people, as the first prerequisite for care was often possession of vital documents and consistent insurance coverage.

### 3.2 Street medicine’s repeated engagement was non-contingent upon adherence and compliance, which strengthened patient self-efficacy and improved treatment adherence.

Participants identified contingent care as one of the most significant barriers to accessing treatment. While contingency often referred to expectations of adherence or compliance, it also encompassed structural and procedural requirements that created barriers for people experiencing homelessness (PEH). For example, treatment plans dependent on laboratory testing, follow-up visits, or other sequential steps often delayed or prevented care. In contrast, non-contingent care - care that was immediate, flexible, and not dependent on meeting prerequisites - emerged as a defining feature of the street medicine approach. This distinction appeared along two primary axes: (1) a patient axis, in which care was not contingent on patient behaviors such as adherence, compliance, or regular attendance; and (2) a clinical axis, in which care was not contingent on prior clinical steps or eligibility criteria, thereby prioritizing immediacy, accessibility, and trust for patients traditionally excluded from care.

Patients described how the street medicine model offered immediate access to care, prioritized patient engagement and retention, and did not impose conditions or require treatment compliance or adherence.

Patients, like Alejandro, emphasized the street medicine team’s commitment to providing non-contingent care - care that was not premised on meeting a set of conditions or on remaining unsheltered. Even after Alejandro transitioned into supportive housing, the team continued to visit and provide services in his new residence.

“They help me regardless of anything. They really care about us out here. I didn’t think they would still see me when I moved over here [transitional housing site], but they do. They always check in with me and help me out when I need it. Before, I had no medical care. Now, I can really see them whenever I need to. I really enjoy all the people who come out to see us. They make it easy to get seen for whatever reason. I’m really happy with them honestly.”

- Alejandro

This approach, which followed patients as they moved through the housing continuum, illustrated a model of care that prioritized continuity and engagement over compliance, thereby fostering long-term trust and sustained participation in healthcare.

Both Carmen and Aliyah emphasized the importance of the team’s non-contingent and non-punitive approach, which recognized the complex realities of their lives and the barriers that made consistent appointment attendance difficult.

“I used to never go. In the beginning, I never went to the appointments he told me about.

Now, I make every appointment. He was never upset with me that I missed appointments. He would tell me I really needed to go. He would keep reassuring me that I needed to do these things in order to get better. So, now, I make sure I do.”

- Carmen

Patients like Carmen emphasized that the team’s unconditional positive regard and willingness to revisit care goals - even after episodes of non-adherence with routine and specialty appointments - fostered trust and psychological safety with the street medicine team. This persistence and acceptance empowered patients to remain engaged in care at their own pace, ultimately supporting more sustainable progress toward health goals and improved long-term adherence.

However, even for patients who were engaged in care, material insecurities shaped circumstances marked by competing interests, such as engaging in care versus survival economies. For example, Aliyah, an unhoused woman experiencing extreme poverty said:

“They addressed everything I needed. I needed to get blood work done, and it got done. They think I might have lupus because it runs in my family. They wanted me to get checked for that. I need to get my blood work done again, that’s not their [the street medicine team’s] fault, though, it’s mine. When you’re on the streets, you have to make money. Maybe it’s for drugs or food. We all have to make money out here to survive. There are things I won’t do for money, so I have to make sure I’m available when I can make money. They scheduled appointments for me; they handled all of them. I needed to make money, so I didn’t go. They will make the appointments and take you to them. A few times, I didn’t show up because I had other stuff I needed to take care of.”

- Aliyah

Even with street medicine, Aliyah internalized her inability to attend appointments as personal failings rather than reflections of systemic barriers rooted in poverty, where survival economies required prioritizing immediate income over consistent healthcare attendance. However, unlike traditional healthcare settings, the street medicine’s commitment to non-contingent care and immediate access led to Aliyah’s retention in care despite her non-adherence even with the provision of care coordination. By approaching care with flexibility and acknowledging her complex, competing priorities shaped by survival economies, the team supported Aliyah in repeatedly re-engaging with her health goals, rather than being excluded from care for non-adherence.

In this way, this health service delivery model provided regular, flexible, and immediate healthcare, operating with the understanding that programs such as street medicine, have the organizational capacity for consistency, whereas PEH often do not. Patients described the linked the continual disruption from encampment sweeps, daily struggles to secure basic needs through survival economies, and the constant work of preserving psychological and physical safety in unsafe environments to their healthcare engagement, or lack thereof. In this way, the street medicine model shifted the burden of consistency from the patient to the model, thereby alleviating associated pressures to “comply” and “adhere,” and increased engagement.

### 3.3 Street medicine integrates harm reduction into medical care to empower and activate patients to engage in health behaviors.

Participants consistently emphasized that street medicine teams operated from a harm reduction framework grounded in humanism, autonomy, pragmatism, and incrementalism. Beyond maintaining a nonjudgmental approach to substance use, providers integrated harm reduction philosophy into all aspects of care delivery, aligning medical and behavioral health practices with patients’ goals and immediate needs.

For example, Alejandro noted that the street medicine team’s harm reduction-based nonjudgmental approach and commitment to humanism and pragmatism reduced his internalized stigma and shame regarding his substance use, creating the conditions for open and honest disclosure of his substance use to providers.

“I’m really honest with them because they don’t judge me or make me feel bad about [using drugs]”

- Alejandro

Unhoused patients who use drugs often face discrimination in traditional healthcare settings, including outright denials of care based on their continued use. In these contexts, substance use becomes both a barrier to treatment and a source of stigma that reinforces shame and negative self-appraisals. In contrast, street medicine teams actively work to counteract these dynamics by embracing the harm reduction principles of humanism and pragmatism - abstaining from moral judgment, accepting patients’ choices, and not requiring abstinence as a condition for care. By reducing stigma and fostering trust, street medicine providers create spaces where unhoused patients feel safe to disclose their needs and challenges, including substance use, and are empowered to engage more fully in care.

Similarly, Aliyah highlighted how the team’s harm reduction approach to her substance use - grounded in nonjudgment and self-determination - enabled her to initiate medication for opioid use disorder (MOUD).

“They were very kind, and they were open to anything. I had to talk about it [using fentanyl], but they didn’t seem bothered by [my drug use]… I was taking the shot (long-acting injectable buprenorphine) out there on the streets. They got me on that. But it’s just too hard on the streets. It’s so hard trying to get sober when you’re on the streets.”

- Aliyah

Although she was unable to sustain adherence while rough sleeping, she emphasized that the team’s commitment to non-contingent care made her feel safe, knowing she would not be discharged from services due to noncompliance. The street medicine team’s commitment to humanism and autonomy allowed her to disclose discontinuation of MOUD without fear of losing support, remain engaged in care, and consider reinitiating MOUD after moving into transitional housing.

Luis described how a harm reduction approach to mental health centering autonomy and pragmatism enabled him to address his depression on his own terms. While struggling with both depression and ongoing substance use, he emphasized that the team’s consistent presence and nonjudgmental, patient-centered approach created stability and empowered him to initiate psychotropic medication. Importantly, the street medicine providers did not assume abstinence from illicit substances or mandatory behavioral health engagement as prerequisites for treatment. Instead, they offered a range of supportive approaches such as psychotropic medication while also supporting linkages to behavioral health services based on Luis’s expressed interest and readiness, integrated harm reduction by prioritizing shared decision making and autonomy into their mental health care delivery.

“I’m being connected to a therapist. I need medications to keep the [psychiatric] disorder leveled, or something, so they are working on getting me a therapist so I can talk about some things… My mind is all over the place, you know. I wasn’t on top of my mental health, and I was really out of it, and ever since I met them, I feel more leveled out. I feel more focused. I feel more like myself and less depressed. The medications they have been giving me have been working really good for me.”

– Luis

For example, Luis, who had long struggled to access psychiatric services, obtained medication directly from the street medicine team. He reported relief from depressive symptoms and, with these initial improvements, developed greater motivation to continue engaging in his behavioral health care.

Robert emphasized that the street medicine provider’s focus on promoting health literacy was rooted in autonomy, individualism, and practiced via shared decision making.

“I never miss an appointment or anything like that because it could be important. Before, I would miss appointments either because I couldn’t make it or because I didn’t think it was important. I’m confident because he explains what you’re going for. He never just says, “Oh, you need to do this and that.” He actually gives you the information that you need, so that way it doesn’t make you so nervous.”

- Robert

This integration of harm reduction into care delivery empowered patients to take a more active role in their care, including engaging in preventive services such as laboratory testing, imaging, by determining the right time based on their emotional, cognitive, and physical resource burdens. By integrating harm reduction principles such as autonomy, individualism, pragmatism, and humanism into the care delivery model, patients experienced increased self-efficacy and healthcare engagement.

### 3.4 Street medicine provides integrated case management to overcome structural disempowerment and facilitate healthcare engagement.

Robert underscored the critical role of care navigation within the street medicine model for enabling healthcare engagement and adherence among unhoused patients. He noted that without consistent access to a phone, many patients are effectively precluded from independently calling to schedule appointments or arranging transportation to reach them. This barrier was compounded by the lack of reliable transit options and the inability to pay for transportation when limited income must be directed toward meeting basic material needs. In traditional systems, these structural barriers make it nearly impossible for unhoused patients to complete even the initial steps required to access care (eClinicalMedicine, 2023). By contrast, street medicine’s integration of care navigation directly addressed these gaps, ensuring that logistical challenges did not become prohibitive barriers to receiving healthcare.

“It’s so much easier having them make appointments for you and figuring out how to get there. Lots of people out here don’t have phones, so it’s not like we can call and make an appointment for ourselves. And if you need a different doctor or something else [specialist care], they can help you get to that.”

- Robert

With the street medicine model assuming responsibility for care navigation, patients like Robert were able to more fully engage in their healthcare and sustain adherence. He described the ease of attending appointments once case managers scheduled them on his behalf, arranged transportation, and coordinated specialist referrals. By explicitly addressing structural barriers as points of intervention, the street medicine approach not only facilitated access but also fostered greater self-efficacy and renewed healthcare engagement among unhoused patients who might otherwise remain excluded from traditional systems.

In traditional healthcare, adherence is treated as the primary marker of success (Martin et al., 2005), yet unhoused patients are held solely responsible for adherence, despite being systematically deprived of the tools and support necessary to do so. Street medicine reoriented this dynamic by treating adherence itself as an intervention point, operationalizing support through service delivery modifications that created the lower-barrier access. This included adapting practices to align with the lived realities of unhoused patients rather than penalizing them for non-compliance. Like Robert, Luis emphasized the importance of care navigation services - such as appointment scheduling, reminders, and transportation coordination - but he also highlighted how the team’s commitment to adherence extended further. The street medicine team delivered his medications directly, provided a pill box to reduce the cognitive burden of timekeeping, and offered reminders to take his medications, demonstrating how adherence can be facilitated through service support in street medicine, rather than enforced as an individual obligation in traditional healthcare.

“With [the street medicine team], they will call and remind me I have blood work today or whatever it is that I am supposed to do that day. That’s what I love about it. I can be very forgetful, and [traditional healthcare setting] don’t let me know I have stuff to take care of. At the clinic, they will tell me when my appointment is, but then I forget, or I can’t find a ride, and it’s hard. [The street medicine team] they are on my case about the stuff or the blood work I need to do, so it’s very helpful when I feel like someone is there to remind me to do it. It can be overwhelming and hard to keep track of if I don’t have that [reminder]. With my heart stuff, I require different doctors, so I need more appointments than someone who probably doesn’t have heart issues. They take care of all of that for me. Any lab work or MRIs I need to do, they schedule the appointment and take me whenever the time comes. They pretty much do everything for me. They remind me about my medications. They drop off pills every week, so I know what I need to be taking. They remind me what they’re for, and I take them. It helps me remember to take the pills every day… They drop off like a box. It’s labeled for each day. That’s something I struggled with. I would either forget to take them, or I would take too many, or something. You wouldn’t believe how hard it is to remember all the medications you are supposed to take. I would go days without taking them because I would forget about them. They do a lot more than anyone else is willing to do. They don’t compare to anyone else because they go beyond what anyone else has done for me. They are always on top of it, and I can always call and talk to them if I haven’t seen them for the day. I think there should be more people like this. I don’t know who else does this stuff.”

- Luis

PEH like Luis often struggled with medication adherence and internalized these challenges as personal failings, rather than attributing these to structural barriers that made it nearly impossible to maintain access to medications or consistently remember when a dose was last taken.

Street medicine reenvisioned the role of adherence in health service delivery from an individual level responsibility to a product of systemic support. Whereas traditional healthcare often weaponized adherence as a gatekeeping mechanism - producing healthcare exclusion and disempowerment - street medicine operationalized a healthcare-first philosophy by embedding care navigation and case management into its service model. Through these interventions, street medicine improved adherence by adapting care practices to the lived realities of unhoused patients, rather than expecting patients to conform to the rigid demands of traditional healthcare systems. This included scheduling and reminding patients of appointments, arranging and covering transportation, coordinating specialist referrals, and delivering medications with supports like pill boxes and reminders - all of which reduced barriers and enabled patients to engage more consistently in care.

## 4. Discussion

Our findings suggest that while the Healthcare First model is conceptually aligned with Housing First through their shared foundation as rights-based approaches, the current study demonstrates how street medicine operationalizes Healthcare First to overcome structural vulnerabilities among PEH. Housing First recognizes housing as a basic human right (Tsemberis, 1999); Healthcare First extends this logic to health, asserting that access to medical and behavioral care is also a fundamental right. Both models emphasize immediate, voluntary, non-contingent access to services - without prerequisites of readiness, abstinence, or strict compliance - and both are grounded in harm-reduction principles that center autonomy, humanism, and pragmatism. In each, responsibility shifts from individuals adapting to rigid systems to systems adapting to lived realities, providing engagement based on need rather than compliance.

In our data, Healthcare First - as enacted through street medicine - was distinguished by three interconnected components. First, patients emphasized that immediate, non-contingent access fostered trust, psychological safety, and sustained engagement. Unlike traditional settings that exclude patients for missed appointments, inconsistent adherence, or ongoing substance use, street medicine delivered care regardless of readiness or compliance, thereby creating conditions for relationship-based continuity rather than episodic, gatekept encounters. Second, teams embedded harm-reduction principles - grounded in humanism, autonomy, pragmatism, and incrementalism - across all healthcare, not only substance use (Hawk et al., 2017). Patients were not penalized for continued use, discontinuation of MOUD, missed visits, or inconsistent medication adherence. Instead, medical and behavioral care were organized to minimize harms, preserve dignity, and support patient-defined goals - managing chronic illness, stabilizing psychiatric symptoms, or reducing substance-related risks. The Healthcare First model parallels the Medication First (MedFirst) model for opioid use disorder treatment, which, like street medicine, provides rapid access to pharmacotherapy and emphasizes immediacy, non-contingency, and patient-centered flexibility as core mechanisms of engagement and retention (Winograd et al., 2020). This approach stands in sharp contrast to “Housing Ready” and traditional clinical models where ongoing use or imperfect adherence often triggers exclusion (Johnson et al., 2012; Omerov et al., 2019; Tsemberis, 1999). Third, integrated care navigation and case management reframed adherence as a system-supported process, not an individual test. Teams scheduled appointments, arranged transportation, assisted with IDs and insurance, provided reminders, and followed up after missed visits. For many participants, this shifted adherence from “impossible without a phone or ride” to achievable with structural support.

These mechanisms directly countered the cycle in which patients internalize systemic failures as personal failures. As one participant (“Luis”) noted, the ease of access and structural support were “unheard of” in his community. With a pill box and reminder system, he could take the correct dose daily - an example of Healthcare First in action, where practical tools and systemic accountability enable adherence rather than punishing lapses.

Even so, Healthcare First and Housing First work in complementary domains. Housing First secures stability and safety but does not by itself guarantee healthcare access; evidence for downstream health outcomes is mixed (National Academies of Sciences, Engineering, and Medicine, 2018; Tsai, 2020; Tsemberis et al., 2004). Healthcare First provides immediate, integrated medical and behavioral care but cannot by itself resolve harms of housing instability. Participants still struggled when encampments were swept or survival needs took precedence. Taken together, Housing First addresses structural violence through stable housing, while Healthcare First - operationalized by street medicine - mitigates health inequities and premature mortality during the period of homelessness, building trust, reducing harm, and sustaining engagement while people await scarce housing resources.

Our results also illuminate how documentation-based and displacement-related barriers perpetuate exclusion from traditional care. State-sanctioned encampment sweeps destroy belongings, including IDs and insurance cards, and insecure sites increase loss or theft (U.S. Government Accountability Office, 2024). Without government identification, patients cannot apply for or renew Medicaid; even with coverage, many clinics require IDs/insurance cards at check-in (National Law Center on Homelessness and Poverty, 2004). In practice, lack of housing itself becomes a structural barrier to care because there is no safe place to store vital documents. Street medicine partially mitigates this by replacing documents, maintaining coverage, and escorting patients through access points - but these are structural failures that ultimately demand structural solutions.

Within traditional healthcare, adherence is often read as motivation. For PEH, structural barriers (transport, storage of belongings, digital portals, fragmented referrals, stigma) routinely derail follow-through and are misread as disinterest, reinforcing exclusion. Participants described how institutions’ limited commitment to harm reduction, documentation requirements, and lack of navigation support translated into self-doubt and disengagement. In contrast, street medicine’s non-contingent, harm-reduction-based delivery - spanning general medical, psychiatric, and substance-use services - created pathways to treatment even when abstinence was not desired and adherence fluctuated. Teams did not require immediate labs or screenings on rigid timelines; instead, they maintained ongoing access, support, and pacing, aligning care with cognitive load, executive function, and survival constraints. This relational continuity fostered trust and sustained engagement.

Finally, these findings clarify how street medicine is not separate from Healthcare First - it is Healthcare First in practice. By embedding harm reduction (Hawk et al., 2017), eliminating contingencies, and absorbing navigation burdens, street medicine creates the structural conditions that allow patients to enact their health goals amid homelessness. Yet its reach has limits: persistent housing shortages, criminalization of encampments, and inadequate income supports require policy-level remedies. Absent these, Healthcare First can mitigate harms but cannot eliminate the drivers of exclusion and premature mortality among PEH.

These findings have critical policy implications. Executive Order (EO) 14321 – Ending Crime and Disorder on America’s Streets signaled a structural retreat from rights-based approaches, replacing Housing First with coercive Housing Ready models that condition access on compliance. This policy reversal undermines decades of evidence demonstrating Housing First’s effectiveness in improving housing stability and health outcomes (Basu et al., 2012; Davidson et al., 2014; Goering et al., 2014; Tsemberis & Gulcur, 2004; Tsemberis et al., 2004; Wolitski et al., 2010). Although EO 14321 does not explicitly target street medicine, it codifies the rejection of harm-reduction and risk-reduction strategies - including distribution of safer use supplies like sterile syringes and pipes - that are foundational to street-based care and integral to Healthcare First’s broader philosophy. Our findings demonstrate that street medicine, as the practical embodiment of Healthcare First, stands in direct opposition to this policy turn. Whereas EO 14321 advances punitive and involuntary interventions that criminalize homelessness, expands civil commitment, and mandates treatment, Healthcare First supports PEH through voluntary, non-contingent access, centering autonomy, dignity, and human rights.

These contrasting frameworks underscore the risks of policy retrenchment: while Healthcare First fosters trust, engagement, and adherence by adapting services to patients’ lived realities, EO 14321 erodes those very conditions through coercion and exclusion. To effectively reduce morbidity, mortality, and social exclusion among PEH, policy must realign to support both Housing First and Healthcare First as complementary, rights-based models that address the structural and health inequities perpetuated by Housing Ready and traditional healthcare systems. Absent this alignment, coercive policies such as EO 14321 will remain not only counterproductive but actively harm-producing, deepening stigma and worsening health inequities among people experiencing homelessness.

### 4.1 Limitations

This study has several limitations that should be considered when interpreting its findings. This study contributes only an initial exploration of street medicine’s perceived impact. Future research is needed to assess intermediate and long-term outcomes, including health engagement, continuity of care, and recovery trajectories among unhoused populations. Second, the sample was small and drawn from two sites in Bakersfield, California, which may limit the transferability of findings to other geographic or service contexts. As is typical of qualitative research, the emphasis was on depth of understanding rather than representativeness, and findings should be interpreted as context-specific rather than broadly generalizable. Additionally, all participants in this study were actively engaged in street medicine services, which may not reflect the perspectives of individuals who are disengaged, distrustful, or less satisfied with this form of care. Their experiences and perspectives remain important areas for future inquiry to better understand the full spectrum of engagement and barriers within street medicine programs.

Furthermore, while this analysis focused on structural vulnerability and patient engagement, we did not specifically examine gendered or intersectional risks within participants’ narratives. However, accounts from participants such as Aliyah highlighted how financial insecurity and gendered vulnerability shaped experiences of structural disempowerment. Existing research shows that unhoused women often engage in survival sex, and these compounded risks intersect to increase exposure to harm. When such vulnerabilities intersect with healthcare encounters, participants may internalize stigma and blame themselves for unmet health needs - rather than recognizing the systemic and material constraints (such as the urgency of addressing withdrawal or basic survival needs) that drive these choices. This dimension warrants further, focused analysis in future studies to more fully understand how gender and intersectionality mediate experiences of care and structural violence.

Future studies should expand on this work by employing larger, multi-site samples and mixed-methods approaches to explore outcomes across diverse settings, while also assessing the scalability, sustainability, and equity impacts of healthcare-first interventions within broader systems of care.

### 4.2 Conclusions

Our findings illustrate that the healthcare first model in its core, commits to immediate access to care, consumer choice, self-determination, harm reduction, and flexible, person-driven supports. Within this framework, care navigation and case management are built into medical and behavioral health services to help patients overcome structural barriers and remain connected to care; however, engagement in these supports is optional, reflecting the model’s commitment to autonomy and choice. We found that, in contrast to conventional healthcare systems that often condition access on compliance or stability, the healthcare first approach removes preconditions for treatment and embeds harm reduction into clinical practice. The conceptual model developed from these findings is parallel to existing Housing first approaches that successfully deliver services to marginalized communities. These findings highlight the urgent need to expand service delivery models, like street medicine, that employ a healthcare-first framework to promote equity, dignity, and improved health outcomes for PEH.

## Supplementary Files

This is a list of supplementary files associated with this preprint. Click to download.

- AppendixBHRJ.docx
- AppendixAHRJ.docx
- AppendixCHRJ.docx

## Data Availability

The datasets generated and/or analyzed during the current study are not publicly available due to the highly sensitive nature of the data, which include participants’ drug use histories, medical information, and narratives about stigmatized and criminalized behaviors. However, de-identified materials may be made available from the corresponding author on reasonable request.

## Abbreviations

MOUD: Medication for opioid use disorder
PEH: People experiencing homelessness
